# Bridging Gaps in Nursing Home Care: Innovations in Palliative Care and Advance Care Planning

**DOI:** 10.1093/geroni/igaf122.151

**Published:** 2025-12-31

**Authors:** Joan Carpenter, Kathleen Unroe, Cari Levy

## Abstract

The 2022 National Academies report The National Imperative to Improve Nursing Home Quality: Honoring Our Commitment to Residents, Families, and Staff and expert consensus panels have identified significant gaps in the delivery of high-quality care for people living with serious illness in nursing homes (NHs). Recommendations to address these gaps include improving NH palliative care (PC) practices and developing strategies for better integration of advance care planning (ACP) into routine care. This symposium highlights research in NHs studying PC and ACP. The first presentation describes the PC knowledge, attitudes, and practices among frontline NH staff (N = 242) in 16 facilities using a validated survey measuring family communication, provider communication, planning/intervention, and bereavement, and patient vignettes. The second presentation illustrates findings of a large qualitative study using a grounded theory approach to develop an empirically derived theoretical model describing how PC is effectively implemented in NHs using interview data from PC and NH staff (N = 33). The third presentation reviews findings from a Delphi study conducted with 18 clinical experts to develop a tool measuring quality of NH-based ACP implementation to guide ACP policy and interventions to facilitate standardized procedures and enhance NH buy-in. The fourth presentation reports outcomes of an embedded pragmatic cluster randomized clinical trial designed to evaluate the implementation of an ACP Specialist Program in 132 nursing homes. The discussant, Cari Levy—a PC and NH expert—will discuss the four presentations implications for advancing PC and ACP practices in the NH setting.

